# Supplementary material for: Availability, accessibility, and impact of social support on breast cancer treatment among breast cancer patients in Kumasi, Ghana: A qualitative study
Source: PLoS One. 2020 Apr 16;15(4):e0231691. doi: 10.1371/journal.pone.0231691 (PMC7162460; doi:10.1371/journal.pone.0231691)
Supplement: Attachment — Submitted filename: Response to Reviewers Social Support and Breast Cancer.docx [file pone.0231691.s001.docx]

**Response to Reviewers**

**The following were the comments from the reviewers and responses indicating how we addressed the comments.**

Review Comments to the Author

1. Reviewer #1: This manuscript is timely and well written. I would recommend a proofreading for grammatical and punctuation issues, i.e., p.4 line 74 has "as well as and it is." Very clear discussion about the cancer options for women in Ghana. The focus on social support is important, especially since there is a lack of awareness about how to manage breast cancer by community members.
   **Response: The word morbidity was left out that made the sentence looked awkward. The word is inserted and the sentence now reads correctly. This is on line 76 in bold. The manuscript was submitted to professional proof reading and editing firm True Editors. It was edited and we strongly feel the manuscript has improved remarkably.**
2. Please provide more detail on why phenomenology was selected. It is appropriate but your audience may need more information about this method in qualitative research.

**Response: We have provided more detail on why phenomenology was chosen and the details can be found on pages 7 and lines 146-152.**

3. The sampling is actually called "convenience" sampling, not convenient (p.7 line 146).

**Response:** **Convenient has been replaced by convenience as the sampling technique and it is on line 154.**

4. You cannot generalize from this sample (see pp.17-18) about how the sample provides data . inconsistent with a quantitative sample. Please revise that section.

**Response: We are not generalizing from this as we have seen different or conflicting findings from other studies. In the conclusion we have made it clear we are not generalizing from this current study. The statement is found on lines 472-475.**

5. In Table 1, please revise the duration of illness (years) elements. The categories need to be discrete, e.g., 0-1, 2-3, 4-5, etc. As they stand the categories are incorrect and presents more than 15 participants.

**Response: The duration of illness in table 1 has been revised and the number of participants is corrected to 15. The table is on page 10.**

6. In the findings section, I would recommend headers about the types of support that emerged from the themes: Informational, Emotional, etc. This information will assist the reader in understanding which theme you are discussing. I might even discuss "food" as an additional theme that people discussed--it appears in the text when discussing healthcare providers and family

**Response: We have provided headers in the findings section as recommended and this can be found as sub-headings under types of social support. These sub-headings include informational, financial/material, food and home chores, emotional, and spiritual support. Under the impact of social support, we have provided reduction in emotional distress and health nutritional choices as sub-headings. Again, under effects of lack of social support, we have provided financial and emotional burden as a sub-heading.**
