# Supplementary material for: Availability, accessibility, and impact of social support on breast cancer treatment among breast cancer patients in Kumasi, Ghana: A qualitative study
Source: PLoS One. 2020 Apr 16;15(4):e0231691. doi: 10.1371/journal.pone.0231691 (PMC7162460; doi:10.1371/journal.pone.0231691)
Supplement: Attachment — Submitted filename: Response to Reviewers Social Support and Breast Cancer.docx [file pone.0231691.s003.docx]

**Response to Reviewers**

**The following were the comments from the reviewers and responses indicating how we addressed the comments.**

1. Reviewer #2: Although the sample is very small, it would be useful to see how many received the various types of social support instead of just a few quotes. There are no quotes from a third of the sample, and many of the quotes come from the same person, so it is hard to get a sense of how widespread the types of support are.

**Response: We have addressed the comment above and added quotes from more participants. We have presented these in bold throughout the findings section of the manuscript for easy location.**

2. The discussion continues to link the availability of social support in this sample to treatment outcome, which is not supported by either this data set or by the literature cited

**Response: We have delinked social support to treatment outcome and this is presented in bold on lines 539-541.**
