# Supplementary material for: Availability, accessibility, and impact of social support on breast cancer treatment among breast cancer patients in Kumasi, Ghana: A qualitative study
Source: PLoS One. 2020 Apr 16;15(4):e0231691. doi: 10.1371/journal.pone.0231691 (PMC7162460; doi:10.1371/journal.pone.0231691)
Supplement: Attachment — Submitted filename: Reviewer Comments for Breast Cancer and Social Support.docx [file pone.0231691.s002.docx]

Reviewer #2: Comments to the Authors:
1. There are still some spelling and grammatical errors.

**Response: We have examined the entire manuscript and cannot pinpoint the exact spelling and grammatical errors through Microsoft spelling and grammar check. We have used Grammarly and found no spelling mistakes. Again, per recommendation from the first review of this manuscript, we submitted the manuscript to a professional editing company and it was edited before we resubmitted the revised manuscript. We would be grateful if the reviewer can help us by pinpointing the pages of line numbers where these are located we can work on the.**
2. The organization of topics could be improved. "Food aid" is addressed in one section, and "Nutritional choices" in another, using the same quote. A quote about being a survivor is listed under "Nutritional choices." A paragraph about informational support is included under the heading of "spiritual support."

**Response: We have looked at the topics again and reorganized. However, the informational support purported be under spiritual support was mistaken. The paragraph containing the informational support comes after the end of statements under spiritual support but not part of it.**
3. The conclusion continues to generalize from this study that social support determines the prognosis and quality of life of breast cancer patients. This study is not designed to answer that question.

**Response: The statement in the conclusion about the generalization has been rephrased and can be found on pages 22 and 23 and lines 480-484.**
4. In the introduction section, the difficulty in a quick and accurate diagnosis of breast cancer in Ghana is attributed to the lack of radiation therapy centers. It is not the function of radiation therapy centers to diagnose breast cancer, but rather to treat it. Diagnosis requires radiology services.

**Response: The statement referenced above in the comment was actually meant to be radiology services capable of diagnosing various cancers as most health facilities in Ghana lack modern imaging technology to diagnose not just breast cancer but other cancers. The statement has been corrected. This is on pages 4-5 and lines 89-93.**
5. There is no clear description of how the sample was chosen. In one place it is described as a convenience sample. In another the authors state that all of the study participants had some experience with social support, indicating that this was an inclusion criterion**.** If so, that would introduce a selection bias. It also contradicts the statement that "A major objective of this study was to find out if breast cancer patients in this study received any kind of support..."It is stated that all of the participants were undergoing breast cancer treatment, yet many of them were several years from their diagnosis. A detailed and clear description of how the sample was chosen is needed.

**Response: Indeed, it was a convenience sample as we located and sampled participants at the Radiotherapy center at KATH. The statement that all participants had experience with social support was not inclusion criterion as the reviewer views it here. The statement was made in reference to the type of qualitative study design, phenomenology, we chose rather than making it an inclusion criteria. The statement has been removed to avoid misunderstanding by readers. Details of the sampling procedure is presented on pages 7 and 8 and on lines 150-171.**

6. There is very little support in the quotations cited for the statement that "participants not only gained more knowledge about breast cancer but were relieved of negative thoughts and were full of hope." There is insufficient data to make that claim.

**Response: The statement reference by the reviewer here was made as inference from the quotations we presented under subsection ‘Reduction in Emotional Distress’. We view that as evidence of gaining knowledge and relieving negative thoughts. This, notwithstanding, we have changed the statement but still believe that participants gained some knowledge and also perceived relief with the social support their received. This is on lines 375 to 377 in bold.**
